# Evaluation of analgesic, anti-inflammatory, anti-depressant and anti-coagulant properties of *Lactuca sativa* (CV. Grand Rapids) plant tissues and cell suspension in rats

**DOI:** 10.1186/s12906-015-0742-0

**Published:** 2015-06-27

**Authors:** Hammad Ismail, Bushra Mirza

**Affiliations:** Department of Biochemistry, Quaid-I-Azam University, 45320 Islamabad, Pakistan

**Keywords:** Analgesic, Anticoagulant, Antidepressant, Anti-Inflammatory, Cell suspension, *Lactuca sativa*

## Abstract

**Background:**

*Lactuca sativa* (lettuce) has been traditionally used for relieving pain, inflammation, stomach problems including indigestion and lack of appetite. Moreover, the therapeutic significance of *L. sativa* includes its anticonvulsant, sedative-hypnotic and antioxidant properties.

**Methods:**

In the present study, the MC (methanol and chloroform; 1:1) and aqueous extracts of seed and leaf along with cell suspension exudate were prepared. These extracts were explored for their analgesic, anti-inflammatory, antidepressant and anticoagulant effects by hot plate analgesic assay; carrageenan induced hind paw edema test, forced swimming test and capillary method for blood clotting respectively in a rat model. The results were analyzed using one-way Analysis of Variance (ANOVA) followed by Turkey multiple comparison test.

**Results:**

Interestingly, the extracts and the cell suspension exudate showed dual inhibition by reducing pain and inflammation. The results indicated that the aqueous extracts of leaf exhibited highest analgesic and anti-inflammatory activities followed by leaf MC, cell suspension exudate, seed aqueous and seed MC extracts. The current findings show that aqueous and MC extracts of seed have the least immobility time in the forced swimming test, which could act as an anti-depressant on the central nervous system. The leaf extracts and cell suspension exudate also expressed moderate anti-depressant activities. In anticoagulant assay, the coagulation time of aspirin (positive control) and MC extract of leaf was comparable, suggesting strong anti-coagulant effect. Additionally, no abnormal behavior or lethality was observed in any animal tested.

**Conclusion:**

Taken together, *L. sativa* can potentially act as a strong herbal drug due to its multiple pharmaceutical effects and is therefore of interest in drug discovery and development of formulations.

## Background

*Lactuca sativa* L. (lettuce) is a leafy vegetable and belongs to the *Asteraceae* family and genus *Lactuca*. It is a valuable dietary source of vitamin K, E and C as well as carotenoids [1]. Traditionally, it is well-known for its use as folk remedy for inflammation, pain, stomach problems including indigestion and lack of appetite [2]. Previously, considerable pharmacological studies have been conducted to evaluate therapeutic significance of the crude extracts of *Lactuca sativa* which showed its anticonvulsant, sedative-hypnotic and antioxidant properties [3]. In addition, anti-inflammatory and anti-nociceptive activities of the seed extracts have also been evaluated [4]. Cell suspension cultures are utilized as appropriate tool for the evaluation of an extensive variety of phenomena, bypassing the structural intricacy of the plant. An efficient method for the regeneration of shoots directly from cell suspensions of lettuce has also been previously described [5]. Moreover, cell suspensions offer appropriate platform for the production of high esteem secondary metabolites and different substances of commercial and economic interest.

So far, no study has been carried out to evaluate the combined effects of *L. sativa* on inflammation, analgesia, blood clotting and depression. Therefore, we have examined the analgesic, anti-inflammatory, antidepressant and anticoagulant effects of leaf extracts, seed extracts and cell suspension exudate of *L. sativa* (CV. Grand Rapids) by hot plate analgesic assay, carrageenan induced hind paw edema test, forced swimming test and capillary method for blood clotting respectively in a rat model.

The hot plate assay is a simple method of the pain reaction in animals by which effectiveness of analgesics can be tested by measuring the heat induced pain response. The application of such stimuli evokes a behavioral response which results in the withdrawal of foot which varies inversely with the intensity of the stimulus. If an increase in latency is observed in the experimental treatment, then the treatment is said to be anti-nociceptive or analgesic. Conversely, if a condition such as an inflammation of the paw, serves to decrease the response latency, it is said to induce hyperalgesia [6]. Inflammation is a condition involving confined increase in the amount of leukocytes and various complex mediator molecules [7]. The most common screening method of acute inflammation has been the prevention of edema in rats by induction of carrageenan. It is believed that the COX (cyclooxygenase) enzyme involved in inflammation can be inhibited by NSAIDs (Nonsteroidal anti-inflammatory drugs) to reduce the edema. But NSAIDs have some side effects like renal and gastric toxicity [8]. Medicinal plants are believed to be cost-effective and harmless source of novel biochemical constituents with strong therapeutic properties.

The forced swimming test (FST), described initially by Porsolt [9], is the frequently used model for evaluating antidepressant property. The basic principle of the assay is that rats ultimately develop immobility when they are released in water containing cylinder after they stop active escape behaviors, like swimming or climbing. During the FST, reduction in the immobility time, delay of its onset and increase of escape activities can be observed after the treatment of antidepressant agents [10].

Anticoagulants play an essential role as mediators for the treatment and prevention of thromboembolic disorders [11]. Due to their pharmacological possessions, plants can serve as the sources for the investigation of new compounds with anticoagulant properties. There is convincing scientific indications representing that the use of phytochemicals with anticoagulant effects and dietary anticoagulants can eventually eliminate or reduce the risks of thromboembolic diseases [12].

The aim of present study extracts and cell suspension culture of *Lactuca sativa* (CV. Grand Rapids) were investigated for their analgesic, anti-inflammatory, antidepressant and anticoagulant effects in rat model. The plant extracts had shown significant activities which represent its significance as potential herbal drug.

## Methods

### Preparation of the extract

The seeds and leaves of *Lactuca sativa* (CV. Grand Rapids) were purchased from local market in Pakistan. The plant was identified by Dr. Muhammad Zafar (taxonomist) in Plant Sciences Department Quaid-i-Azam University (QAU). A voucher specimen (number 128085) was deposited in the “Herbarium of medicinal Plants of Pakistan” in QAU Islamabad, Pakistan. The plant leaves were air dried under shade for five weeks at room temperature. The seeds and leaves were then ground to powder and were macerated in portions of 100 g, with the 250 ml solvent consisting of MC (methanol: chloroform (1:1)) and water (250 ml) for 7 days. The mixture was then shaken thoroughly and filtered with Whatman filter paper 1. Then rotaevaporator was used to concentrate the filtrate. The concentrate was dried at room temperature and these crude extracts were stored at 4 °C for pharmacological studies.

### Preparation of cell suspension culture

Seeds of *L. sativa* were germinated on half strength MS [13] medium after surface sterilization with 15 % sodium hypochlorite for 45 s followed by three times washing with sterile distilled water. Callus was produced by placing the cotyledon explants on MS medium supplemented with 4.4 μM BA and 2.7 μM NAA [13]. After callus generation cell suspension culture was prepared by previously reported method [5].

### Preparation of standard and test drugs

For the experiment, extracts were prepared as 100 mg/ml (10 % DMSO) and rest of the drugs were solubilized in saline (1 mg/ml) and were administered orally in volumes of 1 ml/100 g of rat’s body weight. In this study, one optimal dose (1 g/Kg) was selected which showed maximum effect without any lethal outcome to animals as described previously [4].

### Animals and treatment groups

Adult albino rats of weights between 150-200 g of either sex were used for the study. They were housed in standard aluminum cages and bred with standard diet with water ad libitum in the Primate facility of Faculty of Biological Sciences, Quaid-I-Azam University, Islamabad, Pakistan. The study was legitimated by the Institutional Animal Ethics Committee and all precautions were followed to diminish animal suffering. In each experiment, rats were divided into seven groups having five rats in each treatment and test samples were administered orally in each group.

Group-I: used for negative control in which 10 % DMSO (1 ml/Kg) in distilled water.

Group-II: used for positive control in which activity specific standard drug (10 mg/Kg) was used.

Group-III: cell suspension exudate of *L. sativa* was administered (1 ml/Kg).

Group-IV: Leaf MC extract of *L. sativa* was administered (1 g/Kg).

Group-V: Seed MC extract of *L. sativa* was administered (1 g/Kg).

Group-VI: Aqueous leaf extract of *L. sativa* was administered (1 g/Kg).

Group-VII: Aqueous seed extract of *L. sativa* was administered (1 g/Kg).

### Hot plate analgesic assay

This method is based on induction of pain by heat and was first described by Eddy and Leimbach [14]. DMSO (10 %) was used as negative control while Aspirin (1 mg/Kg) was used as positive control to compare the analgesic effect. Prior to treatment paw licking or jumping response was determined by placing the rats on hot plate set at 55 ± 2 °C and initial reaction time (Ti) was determined by taking the average of two readings. After 30 min of dosage, each animal was engaged on a hot plate and the basal reaction time was obtained by observing jump response or hind paw licking (either appears first) was taken as end point (Tf). The reaction time in seconds was recorded at the interval of 0.5, 1 and 2 h of drug administration with a cut off period of 30 s. Percentage analgesic activity was calculated by the formula:

$$ \mathrm{Percentage}\ \mathrm{analgesic}\ \mathrm{activity} = \kern0.75em \left[\ \left(\frac{\mathrm{Tf}-\mathrm{T}\mathrm{i}}{\mathrm{Ti}}\right) \times 100\ \right] $$

### Carrageenan induced hind paw edema test

*L. Sativa* was evaluated for its anti-inflammatory effect by using carrageenan induced hind paw edema assay [15]. The results were compared with DMSO (negative control) and Diclofenac potassium (positive control). After one hour of dosage, edema was induced by injecting 100 μl of carrageenan (1 % in saline) into the subplanter region of left hind paw. The paw volume was measured immediately before and after the carrageenan injection by using Plethysmometer (UGO Basile 7140) which served as the control readings of paw. Regular interval readings (one hour each) were taken by measuring paw volume up to four hours. The percentage edema inhibition was determined by the formula:

$$ \mathrm{Percentage}\ \mathrm{inhibition} = \kern0.75em \left[\ \left(\frac{\mathrm{X}-\mathrm{Y}}{\mathrm{X}}\right) \times 100\ \right] $$

Where “X” is edema of control rats and “Y” is edema of treatment rats.

### Forced swimming test

The anti-depressant activity of *L. sativa* was determined by the forced swimming test described previously [16]. The rats were enforced to swim inside a vertical cylinder filled with water. Deprived of getting away, the ensuing anxiety produces vigorous swimming action to escape by climbing or diving the walls of the cylinder. When the rats stop all activities except those essential for survival (keeping the head above the water). This was assigned as provoked depression. On the first day rats were placed in the cylinder individually and were forced to swim inside a vertical cylinder (18 cm diameter, 40 cm height and 15 cm holding of water maintained at 25 °C). After 5-6 min the animal becomes stable and stays stationary for almost 80 % of the time. After 15 min in the water the rats were evacuated and permitted to dry before being come back to their home enclosures. This process is called pre-swimming. On the next day, oral dose was given to rats, according to their respective groups as mentioned earlier. DMSO (10 %) was used as negative control and Fluoxetine HCl (1 mg/Kg) was used as positive control. After 30 min of dosage, rats were again placed in the water filled cylinder maintained at 25 °C and the camera was placed to the side of the cylinder. Video recording was started before placing the rats into the cylinder and then the rats were placed in the cylinder and the timer was switched on. After 6 min, recording was stopped; the rats were removed from the water, dried with towels and then placed back to their cages. The water was changed after every 2 rats so that the image produced should be clear and sharp to distinguish the rats behaviors. Using a cumulative stopwatch total immobility time was recorded during the last 4 min of a total 6 min video.

### Anticoagulant assay

Anticoagulant activity is the time required for blood to clot without the presence of any substance. Anticoagulant activity was determined by the capillary tube method as described previously [17]. The tail of the rat was cleaned with spirit and then was pricked by the lancet. The tail was squeezed to obtain a larger drop of blood to be filled into a capillary tube. The time of appearance of the drop of the blood on the cut tail was noted. The capillary tubes were sealed and immersed in water bath at 37 °C. After one minute the tube was taken out and small pieces of the capillary tube were broken at every 10 s until a fibrin thread is seen between the two broken ends. The time interval between the appearance of the blood drop and the thread formation was the clotting time.

### Statistical analysis

The data was analyzed using one-way Analysis of Variance (ANOVA) followed by Turkey multiple comparison test. Results are represented as mean ± S.D. and p < 0.05 is considered to be significant.

## Results and discussion

Here we report the *L. sativa* a herbal drug for its analgesic, anti-inflammatory, anti-depressant and anti-coagulant activities of the seeds and leaf extracts along with the cell suspension exudate. We obtained very interesting and promising results which elucidate the importance of lettuce as a traditional medicine.

### Hot plate analgesic assay

**Fig. 1:**
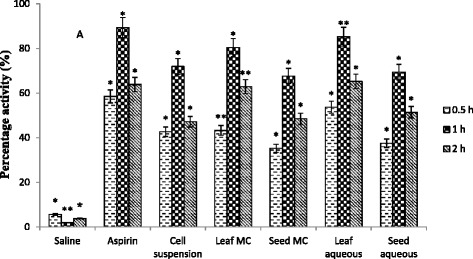
Percentage analgesic effect at different time intervals (Values are expressed in mean ± S.D) where * *p* < 0.05, ** *p* < 0.01 statistically significant as compared to control group

In this study, hot plate assay was used which is one of the most suitable and easy method for the investigation of centrally acting analgesic involving spinal reflexes [18]. The time dependent activity was observed, which became maximum after 1 h as shown in Table 1. Aspirin was used as positive control which exhibited almost 90 % activity (Fig. 1). The results showed that significant activity was observed for all the tested extracts. Among these, leaf extracts were more prominent in reducing analgesia than the seed extracts and cell suspension exudate. The cell suspension exudate showed 71.91 % analgesic activity which was more than the seed extracts as shown in Fig. 1. When comparing the solvent system, it can be concluded that aqueous extracts were more active than the MC extracts. Recently, the presence of flavonoids has been studied in Lactuca species and these flavonoids have been reported to halt prostaglandin synthetase [19]. Since prostaglandins are involved in pain perception. Therefore, it could be proposed that limited accessibility of prostaglandins by flavonoids might be involved in its analgesic effect.

**Table 1:** Latency period in hot plate analgesic assay

Sr. No.	Treatment Groups (n = 5)	Dose	Latency period (sec) ± S.D.			
			0 h	0.5 h	1 h	2 h
Group-I	Negative control (saline)	1 ml/Kg	5.3 ± 0.51	5.6 ± 0.49	5.4 ± 0.55	5.1 ± 0.53
Group-II	Aspirin	10 mg/Kg	9.4 ± 0.61	14.9 ± 0.81**	17.8 ± 0.89**	15.4 ± 0.68**
Group-III	Cell suspension exudate	1 ml/Kg	8.9 ± 0.48	12.7 ± 0.61*	15.3 ± 0.58*	13.1 ± 0.49*
Group-IV	Leaf MC	1 g/Kg	9.7 ± 0.56	13.9 ± 0.78*	17.5 ± 0.85*	15.8 ± 0.64*
Group-V	Seed MC	1 g/Kg	6.8 ± 0.35	9.2 ± 0.28**	11.4 ± 0.62*	10.1 ± 0.71**
Group-VI	Leaf aqueous	1 g/Kg	9.5 ± 0.55	14.6 ± 0.59**	17.6 ± 0.43*	15.7 ± 0.75*
Group-VII	Seed aqueous	1 g/Kg	7.2 ± 0.51	9.9 ± 0.29*	12.2 ± 0.48*	10.9 ± 0.62*

Values are expressed in mean ± S.D. * *p* < 0.05, ** *p* < 0.01 statistically significant as compared to control group

### Carrageenan induced hind paw edema test

**Fig. 2:**
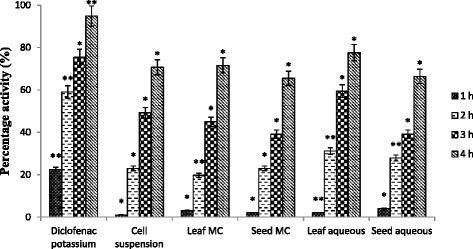
Percentage anti-inflammatory effect at different time intervals (Values are expressed in mean ± S.D) where * *p* < 0.05, ** *p* < 0.01 statistically significant as compared to control group

Carrageenan induced hind paw edema test has been extensively used to evaluate the anti-inflammatory effect of new pharmaceutical agents [2]. All the extracts of *L. sativa* established significant anti-inflammatory effect by regulating biphasic inflammatory process induced by carrageenan (Table 2). The initial phase (1-2 h) of the inflammation is due to the release of serotonin ad histamine while the final phase (3-4 h) is considered by the peak volume of hind paw [20]. As shown in Fig. 2, leaf extracts exhibited maximum percentage inhibition of inflammation. The activity of cell suspension exudate was almost similar to that of a leaf extract in comparison while more than that of seed extract. On the other hand, the results revealed that aqueous extracts were more active in reducing the inflammation than the MC extracts. Our results indicated that oral dose of *L. sativa* repressed the edema preliminary from the 1st hour and throughout all stages of inflammation, which is perhaps due to inactivation of different chemical mediators of inflammation. Phytochemical profile of *Lactuca sativa* suggests the presence of simple phenols, triterpenoids and saponins [4] and triterpenoids are well known for their anti-inflammatory properties [21]. Therefore, it seems that analgesic and anti-inflammatory potential of *Lactuca sativa* seeds might be related to its triterpenoids and saponins.

**Table 2:** Edema volume in anti-inflammatory assay

Sr. No.	Treatment Groups (n = 5)	Dose	Edema volume (ml)^*a*^ ± S.D.			
			1 h	2 h	3 h	4 h
Group-I	Negative control (saline)	1 ml/Kg	0.49 ± 0.02	0.6 ± 0.03	0.68 ± 0.02	1.15 ± 0.02
Group-II	Diclofenac potassium	10 mg/Kg	0.38 ± 0.01**	0.25 ± 0.02**	0.17 ± 0.01**	0.06 ± 0.01**
Group-III	Cell suspension exudate	1 ml/Kg	0.49 ± 0.04*	0.47 ± 0.05*	0.35 ± 0.05*	0.34 ± 0.05*
Group-IV	Leaf MC	1 g/Kg	0.48 ± 0.05*	0.49 ± 0.05*	0.38 ± 0.06**	0.33 ± 0.04**
Group-V	Seed MC	1 g/Kg	0.48 ± 0.08*	0.47 ± 0.08**	0.42 ± 0.06*	0.40 ± 0.07*
Group-VI	Leaf aqueous	1 g/Kg	0.48 ± 0.02*	0.42 ± 0.03*	0.28 ± 0.05*	0.26 ± 0.03*
Group-VII	Seed aqueous	1 g/Kg	0.47 ± 0.08*	0.44 ± 0.05*	0.42 ± 0.07*	0.39 ± 0.08*

Values are expressed in mean ± S.D. * *p* < 0.05, ** *p* < 0.01 statistically significant as compared to control group

^*a*^Difference in initial paw volume and after carrageenan injection

### Forced swimming test

**Fig. 3:**
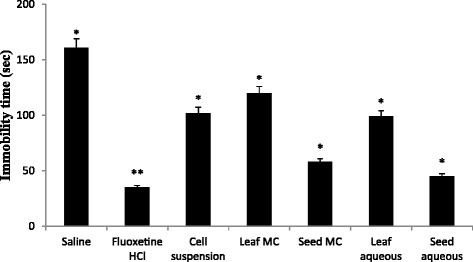
Graphical representation of antidepressant assay (Values are expressed in mean ± S.D) where * *p* < 0.05, ** *p* < 0.01 statistically significant as compared to control group

In the present study, we explored the anti-depressant effect of *L. sativa* via forced swimming test that represents the pharmacological model and produces a state similar to human depression [22]. This test is very specific and sensitive; and the state of depression is decreased by numerous agents like 5HT-reuptake inhibitors, tricyclics and MAO inhibitors [23]. All antidepressant drugs increase the total swimming time and decrease immobility time [24]. It has been recognized that swimming is sensitive to serotonergic compounds for example fluoxetine and that climbing is sensitive to tricyclic antidepressants and drug with selective effects on noradrenergic transmission [23]. The results of antidepressant activity are graphically expressed in Fig. 3. A significant reduction in immobility time was observed for all the extracts when compared with negative control. Similarly, fluoxetine HCl used as positive control showed prominent reduction in immobility time. The immobility by the seed extracts was greater than that of the leaf extracts and cell suspension exudate. It was noted that the immobility activity was comparable in cell suspension exudate and leaf extracts. In literature, it has been reported that treatment with antidepressants reduces the oxidative stress related to depressive disorder and flavonoids contain antioxidant property which is demonstrated experimentally by the rise of the plasma antioxidant status [25].

### Anticoagulant assay

**Fig. 4:**
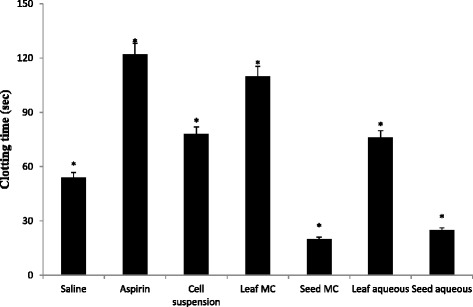
Graphical representation of anticoagulant assay (Values are expressed in mean ± S.D) where * *p* < 0.05, ** *p* < 0.01 statistically significant as compared to control group

The hemostatic mechanisms are meant to arrest bleeding at the site of injury by formation of a hemostatic plug; subsequently there is an eventual elimination of the plug when healing is complete. Normal physiology keeps a delicate balance between these processes and the deficiency or exaggeration of any one mechanism leads to hemorrhage or thrombosis. There are various components via platelets, blood vessels, coagulation factors, plasma inhibitors and the fibrinolytic system, which maintain the physiology [17]. The function of the anticoagulant drugs is to inhibit blood clotting, which is the major cause of heart attacks and strokes [26]. Anticoagulant drugs can be used with a number of diseases when there is a high risk of blood clots. Since anti-coagulants are used for the cardiac problems, hence, instead of relying on blood thinners, physicians can shift to herbal medicine. It has been reported that antioxidants can counteract the hematological and blood coagulation disturbances, oxidative stress, and hepatorenal damages [27]. Our report confirms the anti-coagulant effect of *L. sativa* as it is enriched with the antioxidant constituents and the results are presented in Fig. 4. The mean clotting time of negative control (10 % DMSO) and positive control (Aspirin), which were 54 s and 122 s, respectively, were used as lower and upper limit for the determination of coagulant effect. The most significant activity was shown by the MC leaf extract with clotting time of 110 s. The activity of cell suspension exudate and aqueous extract of leaf was almost similar, representing moderate activity, while the clotting time in seed extracts was less than the negative control indicating their coagulation nature which can be utilized for the treatment of disease like hemophilia.

## Conclusion

The present experimental findings of different extracts suggest that *L. sativa* is a broad spectrum pharmaceutical crop conforming significant analgesic, anti-inflammatory, anti-depressant and anti-coagulant properties that has potential to replace synthetic drugs. More interestingly, cell suspension exudate showed prominent results in all the assays which is the main point of interest because valuable secondary metabolites and economically important substances can be produced in bulk from plant cell suspensions in simple, cost-effective and reproducible way. However, advance study is needed to explore the precise mechanism of action the active components.
